# CCHFV Seroprevalence among Hunter-Gatherers, Northeastern Democratic Republic of the Congo

**DOI:** 10.3201/eid3203.251171

**Published:** 2026-03

**Authors:** Dacquin M. Kasumba, Samuel M. Shamamba, Ange Y. Mubiala, Bobo N. Bazola, Amanda C. Horton, Charles Ndawula, Jean-Jacques Muyembe-Tamfum, Jean-Christophe Sabue Mulangu

**Affiliations:** Institut National de Recherche Biomédicale, Kinshasa, Democratic Republic of the Congo (D.M. Kasumba, S.M. Shamamba, A.Y. Mubiala, B.N. Bazola, J.-J. Muyembe-Tamfum, J.-C.S. Mulangu); Université de Kinshasa Faculty of Medicine, Kinshasa (D.M. Kasumba, J.-C.S. Mulangu); United Kingdom Health Security Agency, Porton Down, UK (A.C. Horton); National Livestock Resources Research Institute, Entebbe, Uganda (C. Ndawula); Global Medical Affairs, Ridgeback Biotherapeutics, Miami, Florida, USA (J.-C.S. Mulangu)

**Keywords:** Crimean-Congo hemorrhagic fever, CCHF, viruses, zoonoses, seroprevalence, pygmy, Democratic Republic of the Congo, DRC

## Abstract

We evaluated human Crimean-Congo hemorrhagic fever virus (CCHFV) seroprevalence in hunter-gatherer populations of northeastern Democratic Republic of the Congo. We tested blood from 300 participants for CCHFV antibodies; 4% were CCHFV-positive. CCHFV likely has been circulating undetected in the country, indicating the need for a more robust surveillance system.

Crimean-Congo hemorrhagic fever (CCHF) is a tickborne viral disease that is endemic to sub-Saharan Africa and has a widespread global distribution (*1*). CCHF virus (CCHFV) belongs to the *Orthonairovirus* genus, in the Nairoviridae family of the Bunyaviridae order (*2*). *Hyalomma* spp. ticks transmit CCHFV through bites, but the virus also can be transmitted to humans via contact with infected blood (*1*). During outbreaks, case-fatality rates can be as high as 60%, and the incubation period is ≈1–6 days, depending on the transmission route. Infections are characterized by a wide range of symptoms, including but not limited to nonspecific fever, myalgia, headache, diarrhea, nausea, and vomiting (3). 

In the Democratic Republic of the Congo (DRC), reported cases have been limited to 2 zoonotic transmissions since 1956 (*4*,*5*). Even so, CCHF cases are likely undetected because of limited surveillance in the country. Furthermore, no serologic evaluation in humans has been reported from DRC. To shed light on CCHFV prevalence in the country, we examined blood samples taken from a pygmy hunter-gatherer population in Watsa, in northeastern DRC, a population that has frequent contact with wildlife (*6*). We recruited 300 participants from 39 different settlements, 150 men and 150 women, whose mean age was 32.2 (SD +14.6) years. Study participants were invited to the study site, where they received informed consent before we administered a questionnaire and collected blood samples. We used a database previously generated as part of a Marburg hemorrhagic fever virus seroprevalence investigation (*7*) (Appendix) to record interview responses and find evidence of CCHFV circulating within the region. 

Using an ELISA assay, we observed a 4% (n = 12) IgG seroprevalence. Most (83.3%, n = 10) of those positive were female; only 2 men were seropositive (odds ratio [OR] = 5.286; p = 0.035). We noted no significant difference in age between the seropositive and seronegative population, and the mean age of seropositive participants did not deviate from mean age of the study population (Table).

**Table:** Characteristics of subjects in a study of CCHFV seroprevalence among hunter-gatherers, northeastern Democratic Republic of the Congo *

Characteristics	CCHFV serology, no. (%)			p value	OR (95% CI)
	Total, n = 300	Negative, n = 288	Positive, n = 12		
Sex					
M	150 (50)	148 (51.4)	2 (16.7)		
F	150 (50)	140 (48.6)	10 (83.3)	0.035	5.286 (1.138–24.549)
Mean age, y +SD	32.2 +14.6	32.3 +14.5	29.9 +17.5	0.649†	0.988 (0.948–1.031)
Professional activity					
Hunting	180 (60)	178 (61.8)	2 (16.7)	0.004	8.091 (1.740–37.616)
Other/not described	120 (40)	110 (38.2)	10 (83.3)		
Animal contact‡					
Rat	216 (72.0)	209 (72.6)	7 (58.3)	0.282	1.890 (0.583–6.128)
Bat	224 (74.7)	215 (74.7)	9 (75)	0.978	0.982 (0.259–3.724)
Monkey	273 (91.0)	261 (90.6)	12 (100)	0.610	0 (0–0.018)

*p value calculated by Fisher exact test, except as indicated. CCHFV, Crimean-Congo hemorrhagic fever virus; OR, odds ratio. †p value calculated by Student *t*-test. ‡>1 animal exposure type possible.

Our investigation questionnaire did not ask whether volunteers were involved in farming. Because pygmies’ subsistence activities do not include farming (*6*), seropositive donors more likely encountered viral vectors in the forest during traditional hunter-gatherer activities. Sociologically, male members of the community are predominantly expected to hunt, bringing home carcasses to be butchered by female members. Among participants, 60% stated they practiced hunting as their primary means of subsistence; the other 40% performed other, unspecified, activities. In contrast to our expectations, nonhunting participants had a higher probability for seropositivity than hunting participants (Table; Appendix Table 1). 

Because most seropositive participants were women who did not routinely hunt, and considering the lifestyle of pygmy populations, we speculate that gathering food or butchering carcasses constituted greater CCHFV risk factors (*1*). However, we cannot exclude the possibility that the nonhunting seropositive female members also undertook farming activities for additional subsistence. Nevertheless, data suggest the possibility that nonfarming activities in the forest could expose humans to CCHFV tick vectors, either through direct interaction in the bushes or via wild animals harboring ticks (*8*–*10*).

To help clarify the risk mediated by exposure via wild animals, we asked participants about animal contacts. Rats, bats, monkeys, and other animals in the area are known to carry ticks responsible for CCHFV transmission (*8*–*10*). We asked participants whether they touched, ate, or had been bitten by animals; 72% reported encountering rodents (particularly rats), 74.7% encountered bats, and 91% encountered monkeys. Although all 12 seropositive participants confirmed previous contact with monkeys, we did not have sufficient elements to statistically confirm that association (OR = 0; p = 0.610) (Table).

Of the 12 seropositive participants, 6 (50%) reported previously having hemorrhagic fever (HF) symptoms; however, we found no significant correlation between seropositivity and HF symptoms (OR = 0.834; p = 0.776). In addition, we found no correlation between seropositivity and direct or indirect contact with another HF patient or a dead body (Appendix Table 2). That finding supports the possibility that CCHFV seroprevalence and transmission was not through direct or indirect human contact within this community, but rather through zoonotic transmission, further supported by the established correlation between reported subsistence activities and seropositivity (Table).

In conclusion, we documented serologic evidence of CCHF in DRC hunter-gatherer populations, indicating that the disease has been circulating undetected in the country. The lack of previously reported cases is probably the consequence of a nonexistent surveillance system. In addition, the lack of specific diagnostic tools in DRC is a challenge to understanding the epidemiology of the disease. Our findings highlight the need for greater scrutiny into risk factors of CCHFV exposure, particularly among populations exposed to wildlife as part of their lifestyles and in regions with diverse human population groups and cultures.

Appendix Additional information on Crimean-Congo hemorrhagic fever virus seroprevalence among hunter-gatherers, northeastern DRC.
