# Supplementary material for: CCHFV Seroprevalence among Hunter-Gatherers, Northeastern Democratic Republic of the Congo
Source: Emerg Infect Dis. 2026 Mar;32(3):447–9. doi: 10.3201/eid3203.251171 (PMC13016016; doi:10.3201/eid3203.251171)
Supplement: Appendix — Additional information on Crimean-Congo hemorrhagic fever virus seroprevalence among hunter-gatherers, northeastern DRC. [file 25-1171-Techapp-s1.pdf]

Article DOI: https://doi.org/10.3201/eid3203.251171

EID cannot ensure accessibility for supplementary materials supplied by authors. Readers who have difficulty accessing supplementary content should contact the authors for assistance.

CCHFV Seroprevalence among Hunter-Gatherers, Northeastern Democratic Republic of the Congo

Appendix

Participants, data collection and blood samples

A population-based cross-sectional study in Watsa was conducted for 5 days, from 12th to 16th August 2002 in the pygmy populations as previously described (*1*). The study population came from 39 different pygmy settlements. Watsa is in the North-eastern part of the DRC and at the time of the survey, its population was estimated to be around 180,000. Study participants were invited to the study site where they were administered informed consent before the questionnaire was administered to them and blood samples collected. Serum from collected blood samples were used to evaluate IgG antibodies raised against CCHFV. Only participants older than 10 years old were eligible. During and after data collection, authors had access to information that could identify individual participants. Retrospectively, archived data were accessed for research purpose and analysis of CCHF serology.

Ethical considerations

The original study was conducted in compliance with the Helsinki Declaration and samples collected in August 2002. Ethical approval was obtained from the ethics committee of the Institute of Tropical Medicine in Antwerp (ITM) (Approval N° MedEth/MP/dvm/637). Verbal informed consent was obtained from each participant before their enrolment into the study (*1*,*2*). Our secondary analysis and publication of anonymized data from the study obtained ethical clearance from the Kinshasa School of Public Health (Approval N° ESP/CE/77B/2025).

CCHFV ELISA

Serum samples collected from each volunteer were tested for the presence of anti-IgG against CCHFV NP antigen by an in-house enzyme-linked immunosorbent assay (ELISA) as described previously (*3*,*4*). Briefly, purified recombinant 96-well plates were coated with either CCHFV NP (diluted 1:2000 in PBS) or mock antigen diluted similarly. The plates were washed and blocked with skimmed milk before diluted (1:100) samples were added in duplicate to well coated with CCHFV NP antigen and mock antigen. Anti-human IgG HRP-conjugated was used for detection. Plates were read at optical density (OD) of 405nm. Net OD values were determined for each sample as follows: net OD = OD in wells with recombinant CCHFV NP antigen minus OD in wells with mock antigen.

Statistical analysis

Statistical considerations were as described (*1*). The interview data were recorded in a database using EpiInfo 6.04 (Centers of Disease Control and Prevention, Atlanta, Georgia, USA), and SPSS Version 27.0 and Prism 8.0. To compare the characteristics of seropositive and seronegative individuals, the χ^2 test and Fisher exact test were used to compare proportions, and Student *t*-test was used to compare means. Odds ratios and exact 95% CIs were used to assess the association between risk factors and CCHFV IgG seroprevalence. The association between age group and anti-CCHFV IgG prevalence was analyzed by the Snedecor Fisher F-test.

References

1. Mulangu S, Borchert M, Paweska J, Tshomba A, Afounde A, Kulidri A, et al. High prevalence of IgG antibodies to Ebola virus in the Efé pygmy population in the Watsa region, Democratic Republic of the Congo. BMC Infect Dis. 2016;16:263. PubMed https://doi.org/10.1186/s12879-016-1607-y

2. Borchert M, Mulangu S, Swanepoel R, Tshomba A, Afounde A, Kulidri A, et al. Pygmy populations seronegative for Marburg virus. Emerg Infect Dis. 2005;11:174–7. PubMed https://doi.org/10.3201/eid1101.040377

3. Samudzi RR, Leman PA, Paweska JT, Swanepoel R, Burt FJ. Bacterial expression of Crimean-Congo hemorrhagic fever virus nucleoprotein and its evaluation as a diagnostic reagent in an indirect ELISA. J Virol Methods. 2012;179:70–6. PubMed https://doi.org/10.1016/j.jviromet.2011.09.023

4. Fisher-Hoch SP, McCormick JB, Swanepoel R, Van Middlekoop A, Harvey S, Kustner HG. Risk of human infections with Crimean-Congo hemorrhagic fever virus in a South African rural community. Am J Trop Med Hyg. 1992;47:337–45. PubMed https://doi.org/10.4269/ajtmh.1992.47.337

Appendix Table 1. Percentage of participants practicing hunting or non-hunting activities in pygmy population in Watsa, DR Congo

	Hunting			
Sex	No	Yes	Total	p value*
Male	0 (0%)	150 (83.3%)	150 (50)	0.00
Female	120 (100%)	30 (16.7%)	150 (50)	
Total	120 (100%)	180 (100%)	300 (100%)	

*By Fisher exact test.

Appendix Table 2. Risks factors associated with potential human-to-human transmission of CCHF in pygmy population in Watsa, DR Congo*

	CCHFV serology test				
Potential risk factors	Total, n = 300	Negative, n = 288	Positive, n = 12	p value†	OR (95% C.I)
Had HF symptoms before?	137 (45.7%)	131 (45.5%)	6 (50%)	0.776	0.834 (0.263–2.649)
A family member had HF symptoms before?	66 (22%)	65 (22.6%)	1 (8.3%)	0.475	3.206 (0.406–25.299)
Worked with or in close proximity with an HF patient?	61 (20.3%)	60 (20.8%)	1 (8.3%)	0.471	2.895 (0.366–22.866)
Shared a house with an HF patient?	47 (15.7%)	46 (16%)	1 (8.3%)	0.699	2.091 (0.264–16.591)
Touched dead body of someone who died of an HF?	57 (19%)	56 (19.4%)	1 (8.3%)	0.474	2.655 (0.336–20.996)
Touched blood, urines, and feces of an HF patient?	34 (11.3%)	33 (11.5%)	1 (8.3%)	0.738	1.424 (0.178–11.383)
Took part in burial rituals of someone who died of HF?	65 (21.7%)	63 (21.9%)	2 (16.7%)	0.668	1.4 (0.299–6.554)
Touched/washed the dead body of someone who died of HF?	44 (14.7%)	42 (14.6%)	2 (16.7%)	0.691	0.854 (0.181–4.034)

*CCHFV, Crimean-Congo hemorrhagic fever virus; HF, hemorrhagic fever.
†By Fisher exact test.
